# An *In Vitro* Comparative Study of Intracanal Fluid Motion and Wall Shear Stress Induced by Ultrasonic and Polymer Rotary Finishing Files in a Simulated Root Canal Model

**DOI:** 10.5402/2012/764041

**Published:** 2012-03-11

**Authors:** Jon Koch, John Borg, Abby Mattson, Kris Olsen, James Bahcall

**Affiliations:** ^1^College of Engineering, Marquette University, Milwaukee, WI 53233, USA; ^2^School of Dentistry, Marquette University, Milwaukee, WI 53233, USA; ^3^College of Dental Medicine Illinois, Midwestern University, Downers Grove, IL 60515, USA

## Abstract

*Objective*. This *in vitro* study compared the flow pattern and shear stress of an irrigant induced by ultrasonic and polymer rotary finishing file activation in an acrylic root canal model. Flow visualization analysis was performed using an acrylic canal filled with a mixture of distilled water and rheoscopic fluid. The ultrasonic and polymer rotary finishing file were separately tested in the canal and activated in a static position and in a cyclical axial motion (up and down). Particle movement in the fluid was captured using a high-speed digital camera and DaVis 7.1 software. The fluid shear stress analysis was performed using hot film anemometry. A hot-wire was placed in an acrylic root canal and the canal was filled with distilled water. The ultrasonic and polymer rotary finishing files were separately tested in a static position and in a cyclical axial motion. Positive needle irrigation was also tested separately for fluid shear stress. The induced wall shear stress was measured using LabVIEW 8.0 software.

## 1. Introduction

No matter which endodontic rotary nickel-titanium (NiTi) file system a clinician chooses to incorporate into their conventional endodontic treatment in conjunction with sodium hypochlorite (NaOCl) and ethylenediaminetetraacetic acid (EDTA) irrigation, there will always be some canal debris left on the dentinal walls [1]. An *in vitro* study by Chuste-Guillot et al. [2] demonstrated, regardless of the NiTi rotary file system used as a root canal preparation technique in the experiment, the root dentin remained infected and was not bacteria-free. There are several main reasons as to why there is residual canal debris after conventional endodontic treatment instrumentation and irrigation. First, nickel-titanium files stay centered in the canal and thus will not contact walls that have various invaginations or irregularities. Second, canal morphology can be complex making it difficult for the chemical-mechanical canal preparation to be effective in removing all the debris.

Current concepts in conventional endodontics recommend the use of lubricating and chelating agents during the cleaning and shaping phase. Also, it has been recommended to use copious irrigation of NaOCl during all phases of instrumentation along with the removal of the smear layer prior to obturation [3]. With apical leakage of bacteria or bacteria toxins from a root canal system being one of the main causes of the prevention of periradicular healing, eliminating as much canal wall debris (smear layer) as possible is important [4]. A study by Ricucci et al. [5] reported that intraradicular infections were the primary cause of endodontic treatment failure. The empirical standard of completion of the chemical-mechanical canal preparation is to work a canal up to the master apical file in conjunction with irrigation of NaOCl and EDTA.

In order to address the remaining canal debris, it is recommended to incorporate sonic or ultrasonic instrumentation along with NaOCl prior to obturation of the canal(s) [6]. The sonic or ultrasonic files used should be no greater than a number 15 or number 20 file in order to prevent instrument binding and allow for optimal instrument performance. The goal is not to further enlarge the canal, but to gently brush the sonic or ultrasonic instrument along the canal walls to remove the remaining debris and agitate the NaOCl into the irregularities of the canal system [7]. Sabins et al. [8] showed that passive sonic or ultrasonic irrigation, for as little as 30 s, resulted in significantly cleaner canals than hand filing alone. The use of ultrasonic irrigation after hand or rotary file instrumentation has demonstrated significantly cleaner canals and isthmuses in mesial roots of mandibular molars [9]. The literature has also reported that there was no significant difference between sonic and ultrasonic instrumentation in removing canal debris after conventional endodontic biomechanical canal preparation [10].

A plastic rotary finishing file, F File (Plastic Endo, LLC, Lincolnshire, IL, USA), was recently introduced into the endodontic instrument market [11]. This single-use rotary finishing file is made of plastic and has diamond abrasive embedded into a nontoxic polymer. Its unique file design enables the removal of dentinal wall debris and agitation of the sodium hypochlorite into areas of the canal that prior instrumentation did not reach, while not further enlarging the canal.

The polymer rotary finishing file is  20 mm at the tip with a 0.04 taper. The plastic rotary finishing file is available in 21 mm, 25 mm, and 31 mm lengths. This tip size and taper provide a better clinical relationship to the rotary nickel-titanium files presently available than do sonic or ultrasonic instruments. An *in vivo* study by West et al. [12] found that there was no significant difference (*P* > 0.05) in residual canal debris removal after conventional endodontic biomechanical canal preparation between a sonic file instrument and an F File. However, there was a significant greater (*P* < 0.01) amount of residual canal debris removed with either sonic or an F File as compared to needle irrigation alone.

The purpose of this *in vitro* study was to compare the fluid flow patterns and shear stress of an irrigant induced by ultrasonic and polymer rotary finishing file activation in an acrylic root canal model, in both static position and cyclical axial motion. Positive needle irrigation was also tested for fluid shear stress analysis.

## 2. Materials and Methods

 The ultrasonic file used was a metal K15 finishing file driven by a Suprasson P5 Booster (Satelec, Bordeaux, France). The polymer rotary finishing file was a size 20/.04, 25 mm F File driven by an AEU-20T Endodontic System (Dentsply, Tulsa, OK, USA) with an Aseptico motor and a 1 : 8 ratio Anthogyr E-type handpiece.

### 2.1. Flow Visualization

**Figure 1:**
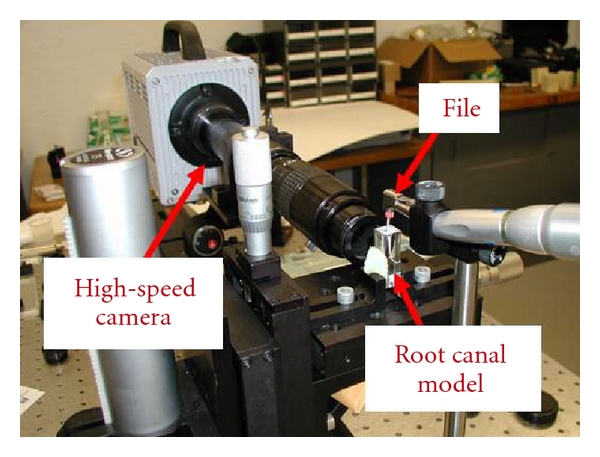
Flow visualization experimental model.

An acrylic root canal model was instrumented to size 30/.06 and clamped to a translation stage to control its location. The model was filled with a mixture of distilled water and rheoscopic fluid. A Photron Fastcam Ultima APX-RS high-speed camera with a Navitar 12x zoom lens and a 5x objective lens recorded images of the fluid motion at a magnification of 4.69x. An ROI 150 Illuminator was used to illuminate the model (Figure 1).

The ultrasonic and polymer rotary finishing files were inserted into the canal and activated in the static position and in a cyclical axial motion (up and down motion at a frequency of *∼*1-2 Hz and a total displacement of 2.5 cm), and images of the fluid motion were taken in each of seven canal sections. The rotary file was rotated at 600 rpm, and the ultrasonic file was operated on setting number 3. Images were recorded at a rate of either 1.5 kHz or 3 kHz. The digital video images were imported into DaVis 7.1 software (LaVision, Goettingen, Germany) and calibrated to enable the analysis of flow patterns through direct observation.

### 2.2. Hot Film

**Figure 2:**
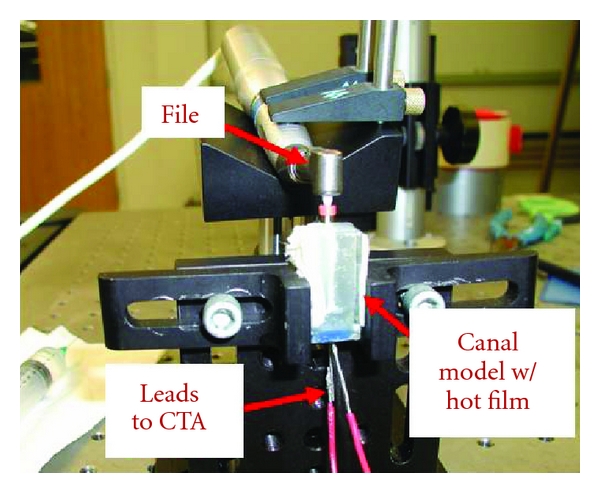
Hot-film experimental model.

 The hot film experiment measured the fluid shear stress induced on the canal wall in one location. Two canal models similar to the one used in the flow visualization experiment were made out of epoxy with a 5 *μ*m hot wire embedded so that the wire was flushed with the canal walls (Figure 2). One model was made with the wire oriented perpendicular to the canal to measure the axial stress, and the other model was made with the wire oriented parallel to the canal to measure the tangential stress. The wire was then attached to a Dantec MiniCTA 54T30 constant-temperature anemometer (CTA) which supplies a voltage to the wire in order to maintain a constant temperature. When the files were rotated or vibrated, they induced fluid motion which cooled the wire and caused an increase in the supplied voltage.

 The canal models were filled with distilled water, and the ultrasonic and polymer rotary finishing files were inserted separately into the canal and activated in the same way as for the visualization experiments. Data for the ultrasonic and rotary files were recorded at rates of 300 and 1 kHz, respectively. Data was also collected at 1 kHz while irrigating the canal with distilled water using a 30G needle in order to compare the shear stress induced from positive pressure irrigation alone. Voltages were sent from the CTA to a National Instruments BNC-2110 DAQ device and recorded using LabVIEW 8.0 software (National Instruments, Austin, TX, USA).

Time and voltage offsets were applied to the data so that each trial was synchronized. A temperature correction was also applied to the ultrasonic data to account for the rising temperature of the file and the water during operation, as measured via a thermocouple. The voltages were converted to shear stress using calibrations made by applying a known shear stress to both of the wires and measuring the corresponding voltage [13].

## 3. Results

### 3.1. Flow Visualization

#### 3.1.1. Polymer Rotary Finishing File

The flow induced by the polymer rotary finishing file in the static position was laminar because the flow pattern repeated periodically throughout the operation of the file with little variation. The fluid rotated circumferentially with the file while also oscillating axially along the canal. The axial flow was caused by two forward-facing steps on opposite sides of the file. The interaction between the forward facing steps and the wall of the canal created a helical gap which opened and closed twice per revolution and caused the fluid in the gap to move axially along the canal wall.

**Figure 3:**
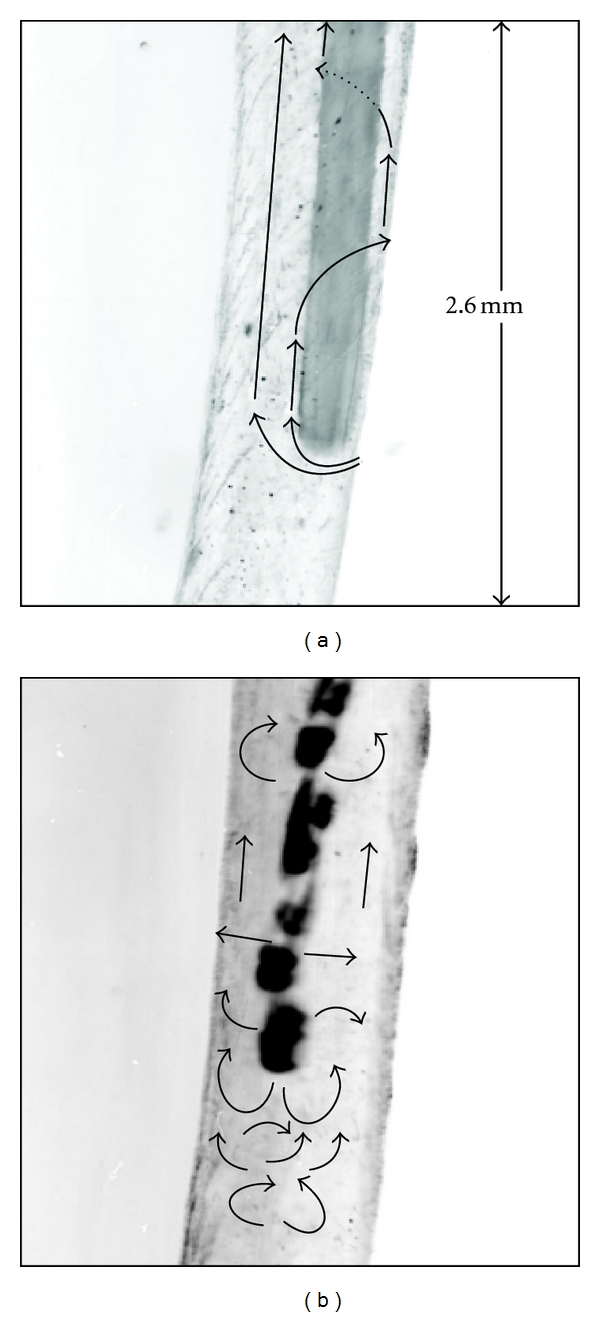
Flow patterns induced by (a) the rotary file and (b) the ultrasonic file. Region shown is centered 8.7 mm from the bottom of the canal.

The fluid motion induced by the polymer rotary finishing file in clinical motion is shown in (Figure 3(a)). The flow field for the moving rotary file was also repeatable and laminar. The fluid followed the same flow pattern as it did with the static file, except that a large translational motion along the axis of the file was superimposed due to the displacement of the fluid by the file. The fluid rotated in an upward and downward spiraling motion around the file as the file moved into and out of the canal, respectively. As the file moved in and out of the canal, it became bound against the wall several times but still continued to rotate.

#### 3.1.2. Ultrasonic File

The flow induced by the ultrasonic file in a static position was transitional because the flow pattern was not repeatable, but large-scale structures were visible. The dominant features in the flow from the ultrasonic file were periodic cells formed by jets emitted from the file and zones of recirculation also shown in a study done by Ahmad et al. [14]. Some mixing between the recirculating cells was seen to occur.

The flow field for the moving ultrasonic file is shown in Figure 3(b). Because of its thinner diameter, there was not much axial motion induced by the movement of the file in comparison to the rotary file. From when the file first entered the canal until, it was about a third of the way into the canal, was in its most unrestrained state, and created a lot of fluid motion. However, as the file moved further into the canal, it became bound against the wall several times, at which point the file stopped oscillating and the fluid motion ceased completely.

### 3.2. Hot Film

#### 3.2.1. Hot Film Shear Stress Measurements

**Figure 4:**
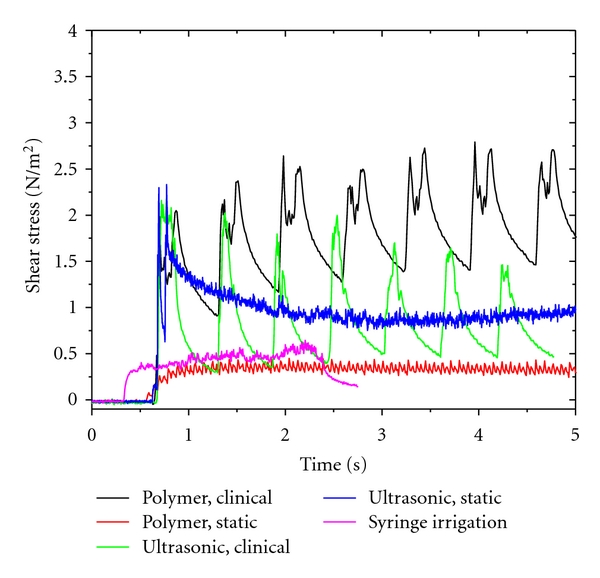
Shear stress results from hot-wire measurements of the ultrasonic and polymer files in clinical motion and in static position. Syringe positive pressure irrigation is also shown for comparison.

Figure 4 shows typical shear stress measurements obtained from the four operating conditions under investigation. In addition the shear stress for the positive aspiration syringe irrigation is presented for comparison. In all cases, the measured shear stress did not exceed 4.0 N/m^2^ at any time. The mean fluid shear stresses were estimated for the ultrasonic and polymer rotary finishing file in static positions during steady-state operation. For the ultrasonic file, this was between 3 and 4 seconds in time as shown in Figure 4. For the polymer file, and for needle aspiration, this was between 1 and 2 seconds on Figure 4. The mean ultrasonic shear stress was 0.86 N/m² while the mean polymer shear stress was 0.34 N/m². Positive needle aspiration wall shear stress was 0.46 N/m². The three data sets were compared using a *t* test and found to be significantly different during the steady-state period (*t*-test, *P* < 0.05).

In clinical motion the measured stress oscillated in phase with the motion of the tool at 1-2 Hz as shown in Figure 4. Clinical motion caused the shear stress generated by the polymer file to vary between 0.9 N/m^2^ and 2.8 N/m^2^ (min to max). From flow visualization and knowing that the stress is maximized when the velocity gradient is maximized, the maximum shear stresses occurred during file movement when the file was near its fully inserted position. This is due to the pumping action of the instrument itself. As the instrument is inserted into the canal, it displaces the fluid and induces an axial flow. This axial flow in turn induces additional shear stresses on the canal wall. Thus, the clinical motion increased the shear stress to a maximum of 2.8 N/m^2^. Clinical motion caused the shear stress generated by the ultrasonic file to vary between 0.3 N/m^2^ and 2.2 N/m^2^ (min to max). In this case, the shear stress maximum occurred for a slightly different reason. Flow visualization indicated that the highest fluid velocities were generated at the lower end of the file. Hence, the maximum shear stress at the hot film location was likely generated when the file tip passed near the hot film.

While differences in the mean shear stresses between the files are significant in the static position, there is measurement error in the magnitude of the shear stresses reported due to the calibration of the hot film. The calibration resulted in a linear relationship between the voltage measured and the applied shear stress with a sensitivity of 0.18 V/(N/m^2^). However, the uncertainty in the known applied shear stress was ±60%. In addition, the wire heating effect was detrended from the data with a maximum correction in the voltage of 0.38 V. For a nominal sensitivity of 0.18 V/(N/m^2^), the temperature correction corresponds to a possible error of 0.07 N/m^2^ or 39%. We thus estimate the absolute accuracy of the shear stress measurements as within a factor of two. The relative change in stress between the files and the syringe, however, is not affected by this uncertainty since the same models, hot wires, and calibrations were common to the three instruments tested.

It is well known that increases in temperature can exponentially increase chemical reaction rates [15, 16]. It is important to note that concomitant experiments done using fine thermocouples resulted in the detection of significant heating of the water when the ultrasonic file was used, but no measureable heating was detected with the polymer file (<0.1°C). Thermocouples to measure temperature were placed in two locations: 4 mm from the lower end of the canal at the same location as the hot film and at the open end of the canal just below the irrigant level. The increase in average temperature measured for the first ten seconds of ultrasonic activation was approximately 3°C, 4°C, and 5°C for power settings 1, 2, and 3, respectively.

## 4. Discussion

The results of this study demonstrated that the polymer rotary finishing file induced a laminar fluid flow pattern, and the ultrasonic file induced a transitional fluid flow pattern in both the static position and cyclical axial motion. The total mean fluid shear stress for the ultrasonic and polymer rotary finishing file in a static position was 0.90 N/m² and 0.34 N/m², respectively, and in cyclical axial motion was 0.77 N/m² for the ultrasonic file and 2.20 N/m² for the polymer rotary finishing file. Positive needle aspiration fluid shear stress was 0.54 N/m².

Laminar flow is typically described as a sheet-like or layered pattern of fluid movement with little mixing between the layers. Transitional flow, an intermediary stage, is characterized by a mixture of both laminar and the more irregular, random fluid movement of turbulent flow [17].

The magnitude of the shear stresses induced by the ultrasonic file observed in this experiment was much smaller than previous estimates of peak shear stress reported by Lumley et al. [18]. However, previously reported estimates were based on a model that estimated shear stress in a different location—at the tip of and surface of the file.

Hot-film anemometry is a highly evolved technique in engineering applications for measuring fluid velocity and shear stress [19, 20]. Hot-wire anemometry has been used to characterize the acoustic streaming velocity and shear stress induced by ultrasonic beam systems, similar to those produced by an ultrasonically activated endodontic file [21]. The current experimental model measurements represent first estimates of fluid shear stress levels at the canal wall. Stress levels measured at the canal wall are so low as to categorize mechanical cleaning by the fluid as likely an insignificant contributor to the debridement process, on a par with the forces exerted by irrigation alone.

The polymer rotary finishing file created a larger amount of fluid shear stress in clinical motion than in a static position because the fluid was displaced by the file forcing rapid fluid flow through the small gap between the file and the canal wall. For the smaller ultrasonic file, the gap is much larger between the instrument and the canal wall, and thus the shear stress caused by fluid displacement is not observed. During clinical motion, the ultrasonic file would periodically bind against the canal wall. The flow visualization experiment demonstrated that when the ultrasonic file became bound it stopped oscillating causing the fluid motion to stop almost instantly. This may be responsible for the ultrasonic file's decrease in average shear stress during clinical motion.

Positive needle aspiration shear stress was 0.54 N/m². In the experimental model, the lumen of the syringe was directly aimed at the hot wire imbedded in the acrylic root canal model. Boutsioukis et al. [22] demonstrated that the highest velocity of fluid flow was within the irrigating needle lumen. Fluid velocities then dropped by an order of magnitude as the fluid exited the lumen because of the sudden expansion of the area downstream of the needle outlet.

Although the polymer rotary finishing file fluid shear stress force was quantitatively greater than the other groups tested, clinically the fluid shear stress observed from the static position and cyclical axial motion with both the ultrasonic and polymer rotary finishing file along with the positive pressure needle irrigation would not be clinically powerful to remove residual canal debris alone. These reported shear stress values are equivalent to the force of a piece of loose leaf paper wiping the dust off of a desk top. Therefore, it is the fluid shear stress, the physical contact of the agitation instrument along the canal wall, and chemical irrigants when combined that are key clinical factors in removing residual canal debris after conventional endodontic biomechanical treatment.

In an *in vitro* study by Townsend and Maki [23], they found no significant differences between the use of ultrasonic agitation and the use of the EndoActivator, F File, and sonic agitation in removing bacteria, but did report that ultrasonic agitation was significantly more effective in removing bacteria than needle irrigation (positive pressure) alone and the EndoVac (negative irrigation pressure).

The effectiveness of chemical irrigants in combination with agitation to remove residual canal debris is supported by Kuah et al. [24]. They demonstrated a one-minute use of ETDA used in conjunction with ultrasonic irrigation and followed by a final flush of NaOCL had significantly more specimens with complete smear layer and debris removal as compared to EDTA irrigation alone. Chopra et al. [25] also demonstrated that the most effective treatments in removing smear layer were the usage of the F File or ultrasonics with NaOCL irrigation in combination with EDTA.

Distilled water was used as the intracanal fluid in the flow visualization experiment because it mixed well with the rheoscopic fluid that allowed the digital camera to detect fluid motion. Distilled water was used in the hot wire experiment because it would not corrode the wire. It is important to note that distilled water has similar density (998 kg/m³) as compared to EDTA (1100 kg/m³) and a similar fluid viscosity (distilled water 0.001003 Kg/m-s) to NaOCL (0.00111 Kg/m-s).

Cavitation is defined as the rapid vaporization and recondensation of a liquid as it briefly flows into a region of low pressure from a region of high pressure. This low-pressure space is made up of water vapor that collapses or implodes upon itself unevenly from the high-pressure fluid moving into this space causing a jet of water to create shock waves that are often extremely forceful [26]. In this experiment, there was no evidence that a cavitation effect was caused by the ultrasonic or polymer rotary finishing file. It is important to note from an endodontic perspective that cavitation cannot be seen by the naked eye and it happens in milliseconds of time. Also, if it were to occur from endodontic instruments, the cavitation would be adjacent to the instrument and not at a distance. Lastly, bubbles are not a clinical sign of cavitation but rather a sign of chemical reaction.

## 5. Conclusions

The results of this study demonstrated that the polymer rotary finishing file induced a laminar fluid flow pattern and the ultrasonic file induced a transitional fluid flow pattern in both the static position and cyclical axial motion. The total mean fluid shear stress for the ultrasonic and polymer rotary finishing file in the static position was 0.90 N/m² and 0.34 N/m², respectively, and in cyclical axial motion was 0.77 N/m² for the ultrasonic file and 2.20 N/m² for the polymer rotary finishing file. Positive needle aspiration shear stress was 0.45 N/m². Although the polymer rotary finishing file fluid shear stress force was quantitatively greater than the other groups tested, clinically the fluid shear stress observed from the static position and cyclical axial motion with both the ultrasonic and polymer rotary finishing file along with the positive pressure needle irrigation would not be clinically powerful enough to remove residual canal debris alone after conventional endodontic chemical-mechanical treatment.
